# Early wound reactions of Japanese maple during winter dormancy: the effect of two contrasting temperature regimes

**DOI:** 10.1093/aobpla/plu059

**Published:** 2014-09-30

**Authors:** Paul Copini, Jan den Ouden, Mathieu Decuyper, Godefridus M. J. Mohren, Antoon J. M. Loomans, Ute Sass-Klaassen

**Affiliations:** 1 Forest Ecology and Forest Management Group, Wageningen University, PO Box 47, 6700 AA Wageningen, The Netherlands; 2 Netherlands Food and Consumer Product Safety Authority, National Plant Protection Organization, PO Box 9102, 6700 HC Wageningen, The Netherlands

**Keywords:** *Acer palmatum*, Japanese maple, local xylem growth, temperature, winter dormancy, wound reactions.

## Abstract

During winter dormancy, temperate trees are capable of only a restricted response to wounding. Depending on the ambient temperature during winter dormancy, wounded trees may start compartmentalization, e.g. by producing inhibitory compounds, but it is thought that processes involving cell proliferation, such as the formation of callus and wound xylem, are delayed until the next growing season. We investigated the effect of two contrasting temperature regimes on early reactions of *Acer palmatum* trees to wounding during winter bud dormancy. Stems of *A. palmatum* trees were wounded and stored under an ambient temperature of 4 or 15 °C for 3 weeks during winter bud dormancy. We then studied wound reactions in the living bark, cambial region and xylem. In the 4 °C treatment, wound reactions were virtually absent. In the 15 °C treatment, however, trees reacted to wounding by dieback of the cortex and phloem and by the formation of ligno-suberized layers. In the cambial zone, cambial dieback occurred and callus tissue and wound xylem were formed locally, close to the wound margins. In the xylem, compartmentalization took place by deposition of inhibitory compounds in fibre cells and vessel elements. We conclude that temperature is an important factor in wound reactions during winter dormancy, and may even induce proliferation of callus and wound xylem within a 3-week period. It therefore seems likely that trees that have been wounded during dormancy in areas with mild or warm winters might cope better with wounding, as unlike trees in cold environments, they may compartmentalize wounds even during winter dormancy.

## Abstract (precis)

During winter dormancy, temperate trees are capable of only a restricted response to wounding. In an experiment, we investigated the effect of wounding on *Acer palmatum* trees during winter-bud dormancy and found that in the cold (4 °C) temperature treatment, wound reactions were virtually absent. In the warm (15 °C) treatment, however, trees reacted actively to wounding within a three-week period by e.g. forming callus and local wound xylem. We conclude that temperature is an important factor in wound reactions during winter dormancy and may even induce the formation of callus and wound xylem within a three-week period.

## Introduction

Trees have evolved effective defence mechanisms to protect their physiologically active xylem and phloem after wounding (Shigo 1984; Pearce 1996; Frankenstein *et al.* 2005, 2006; Deflorio *et al.* 2009). Whatever be the size or nature of the wound, the tree tends to react by forming boundary layers to compartmentalize the wound (Shigo 1984; Bostock and Stermer 1989; Pearce 1996; Deflorio *et al.* 2009). When the living bark (cortex and phloem) is wounded, cells directly adjacent to the wound release inhibitory compounds and then die. Cells further away start forming a ligno-suberized layer, after which a wound periderm develops (Biggs 1986; Oven *et al.* 1999; Renzi *et al.* 2012). If the cambial zone is affected by wounding, cambial cells around the wound die and adjacent intact cells react by forming callus tissue (traumatic parenchyma cells) and wound xylem to overgrow the wound (e.g. Schmitt and Liese 1992; Grünwald *et al.* 2002; Stobbe *et al.* 2002; Dujesiefken *et al.* 2005; Frankenstein *et al.* 2005; Copini *et al.* 2014). If wounds reach into the sapwood, parenchyma cells secrete inhibitory compounds, and distinctly coloured boundary layers start to form in the axial, radial and tangential directions (Shigo 1984; Schmitt and Liese 1992; Shortle *et al.* 1995; Pearce 1996; Dujesiefken *et al.* 2005; Deflorio *et al.* 2009). In the discoloured zone, vessel elements may be blocked by tyloses, or by secretion of inhibitory compounds known as vessel plugs or gels (Murmanis 1975; Bauch *et al.* 1980; Schmitt and Liese 1992).

Wound reactions are temperature dependent and therefore there are clear differences between reactions that occur during the growing season and those occurring during winter dormancy (Murmanis 1975; Armstrong *et al.* 1981; Shigo 1984; Dujesiefken *et al.* 1991, 2005; Schmitt and Liese 1992; Barnett and Miller 1994; Liese and Dujesiefken 1996; Copini *et al.* 2014). In temperate deciduous hardwood trees, wound reactions that entail cell dedifferentiation and cell proliferation (such as the formation of wound periderms, callus cells and wound xylem) occur only during the growing season (Trockenbrodt 1991; Grünwald *et al.* 2002; Frankenstein *et al.* 2005; Copini *et al.* 2014). In addition, whereas inhibitory compounds begin to be deposited in the xylem and living bark soon after wounding during the growing season (Schmitt and Liese 1993; Fink 1999; Grünwald *et al.* 2002; Frankenstein *et al.* 2005; Copini *et al.* 2014), if this reaction occurs after wounding during winter dormancy, smaller amounts of compounds are involved and the deposition tends to be restricted to the wound margins (Jurásek 1958; Murmanis 1975; Schmitt and Liese 1992; Copini *et al.* 2014). Regardless of the season of wounding, the cambium around the wound usually dies back; dieback tends to be more severe if the wound was incurred during winter dormancy (Smith 1980; Dujesiefken and Liese 1990; Dujesiefken *et al.* 1991, 2005; Copini *et al.* 2014).The development of cambial dieback over time has hardly been studied. So far, it has been shown that cambial dieback occurs within 2 weeks shortly before, during and just after the end of the growing season (Copini *et al.* 2014).

Here we report on an investigation of early wound responses of Japanese maple trees (*Acer palmatum* Thunb.) that were wounded and then exposed to a short period of mild temperature during winter dormancy. Japanese maple is native to Japan, Korea and China and is exported as an ornamental tree (e.g. to the USA and Europe). During export or storage and after planting in temperate or subtropical climates, the trees may experience contrasting temperatures during winter dormancy (Copini *et al.* 2010). We hypothesized that wound responses entailing cell proliferation such as formation of callus, wound xylem and wound periderms are absent during winter dormancy (Fink 1999; Begum *et al.* 2007, 2013), while cambial dieback and deposition of inhibitory compounds followed by discolouration may occur during winter dormancy (Schmitt and Liese 1992; Copini *et al.* 2010).

## Methods

### Plant material

We used 20 red-leaved Japanese maples (*A. palmatum* ‘Bloodgood’ Thunb.) with stem diameters of 3.2 ± 0.2 cm (mean ± standard deviation, *n* = 10) in 2010 and 4.2 ± 0.7 cm (*n* = 10) in 2011 at ∼30 cm stem height. The average tree height was 135 ± 15 cm in 2010 and 149 ± 19 cm in 2011. The trees had been grown at a local nursery, where they had been planted in pots in 2009, when the trees were ∼5 years old. In March 2010, the potted trees were placed in a 2 × 2 m grid in an experimental garden in Wageningen, the Netherlands (51.9884°N, 5.6644°E). The trees were watered with a semi-automatic fertigation system.

### Experimental setup

Ten trees were wounded by inserting a 1-mm diameter nail ca. 1 cm into the stem at ca. 30 cm stem height on 20 December 2010, ∼2.5 months after leaf shedding. Directly after wounding, five randomly selected trees were placed in a dark climate chamber at 4 °C; the remaining five were also stored in a dark climate chamber, but at 15 °C. On 10 January 2011, a stem segment ∼10 cm long that included the wound was cut from each of the 10 trees and stored in a 50 % ethanol solution at 4 °C. One year later, between 20 December 2011 and 10 January 2012, a new batch of 10 trees was subjected to identical treatments, using identical climate chambers.

### Sample preparation

All 20 stem sections were sawn transversally through the middle of the wound. Using the G.S.L.-1 sliding microtome (Gärtner *et al.* 2014) we then cut transverse thin sections (20–25 μm) through the wounded part. All cross-sections were stained with a safranin/astra-blue solution for 5 min, to colour unlignified cells blue and the lignified cells and cells filled with inhibitory compound red (Gärtner and Schweingruber 2013). Additional samples were immersed in potassium hypochlorite (5 %) for ca. 8 min, then rinsed with water and stained with (i) a safranin/astra-blue solution to stain unlignified cells blue and lignified cells red (Gärtner and Schweingruber 2013) or (ii) Sudan III, in order to indicate suberine. Following dehydration in graded series of ethanol (50–95–100 %), all samples were rinsed with xylol, mounted on microscope slides in Canada balsam and dried in an oven at 60 °C for 15 h. Photographs were taken with a digital camera (DFC 320, Leica, Cambridge, UK) mounted on a microscope (DM2500, Leica), using Leica imaging software (version 3.6.0).

### Measurements and statistical analyses

**Figure 1.**
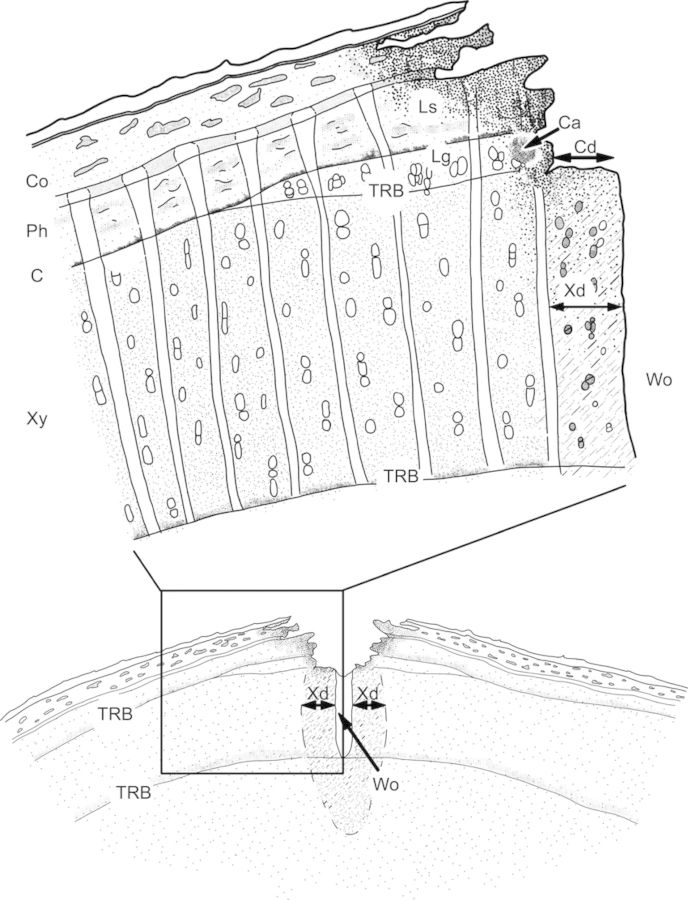
Schematic overview of the anatomical features which occur after wounding (Wo, wound) and were recorded or measured in the phloem (Ph), cortex (Co), cambial region (C) and xylem (Xy). In the living bark, i.e. phloem and cortex, we recorded the presence of ligno-suberized layers (Ls) and wound periderms. In the cambial zone, we measured the extent of tangential cambial dieback (Cd) and recorded the presence of callus (Ca) and local growth (Lg) of wound xylem, which develops after the TRB has been formed. In the xylem, we measured the extent of tangential xylem discolouration (Xd) left and right of the wound (Wo).

We examined all the treated thin sections and recorded the presence or absence of the formation of ligno-suberized layers and wound periderms in the living bark (Fig. 1). All other analyses were based on thin sections that were stained with safranin/astra-blue solution and not treated with potassium hypochlorite. In the cambial zone, we measured the extent of tangential cambial dieback as the distance between the wound and the intact cambium (Fig. 1), using the software Leica Application Suite (version 3.6, Heerbrugg, Switzerland). In addition, the presence of callus tissue (traumatic parenchyma cells) and the locally present wound xylem were recorded after the outermost tree-ring boundary (TRB) had been located (Fig. 1). We used Leica Application Suite software (version 3.6) to determine the mean tangential width of xylem discolouration from measurements taken left and right of the wound (Fig. 1). Differences in wound response between the warm and cold treatments were tested per winter with the statistical software package SPSS version 19 (SPSS, Inc., Chicago, IL, USA), applying a significance level of 0.05. The effects of temperature on cambial dieback and xylem discolouration were analysed using the non-parametric Mann–Whitney *U*-test; the presence of ligno-suberized layers, callus and wound xylem was analysed using Pearson's Chi-square tests.

## Results

All buds remained dormant during both the 3-week experiments (winters of 2010/2011 and 2011/2012).

**Figure 2.**
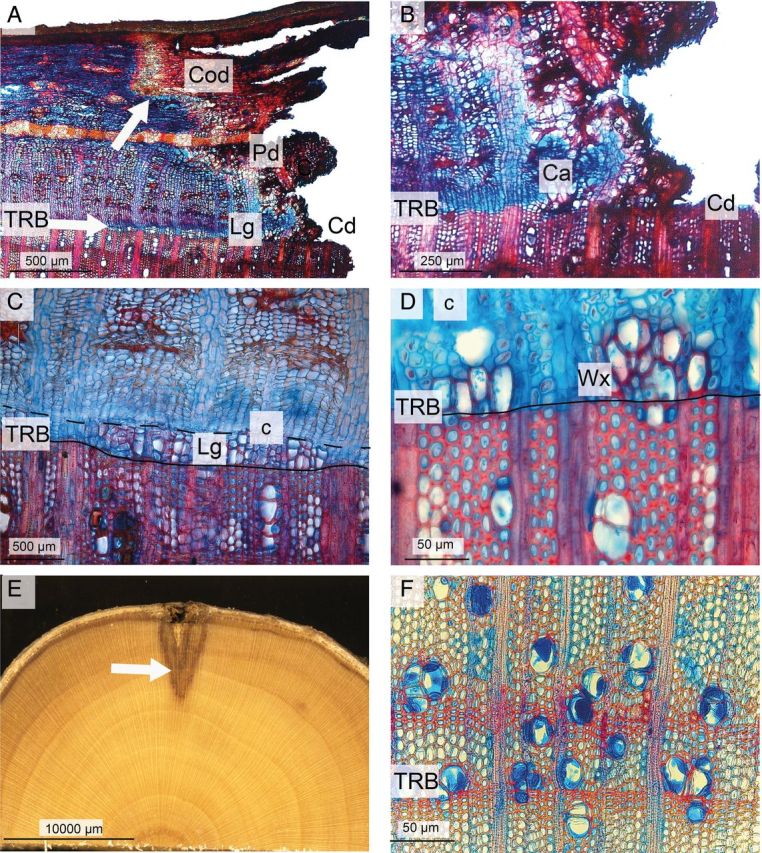
Wound reactions of dormant *A. palmatum* after being stored for 3 weeks in a climate chamber at a temperature of 15 °C. (A) Thin transverse section showing Cd and Lg resulting from the formation of wound xylem to the right of the lower arrow. The upper arrow indicates the ligno-suberized layer that is developing between the area with phloem discolouration (Pd), cortex discolouration (Cod) and the living phloem and cortex, to compartmentalize the wound in the phloem and cortex. (B) Thin transverse section showing callus formation (Ca) in relation to the TRB and Cd. (C) The reactivated cambium has formed wound xylem locally near the wound margin in between the TRB (solid line) and the cambium (c) above the dashed line. (D) Partly lignified and unlignified vessels. Fibre cells and ray parenchyma are still unlignified below the cambium (c). (E) Transverse section through the wood and the bark surrounding the wound; the arrow indicates xylem discolouration produced by secretion of inhibitory compounds. (F) Transverse section showing secretion of inhibitory compounds into vessels and fibre lumen close to the intact xylem, as indicated by the arrow in Fig. 2E.

**Figure 3.**
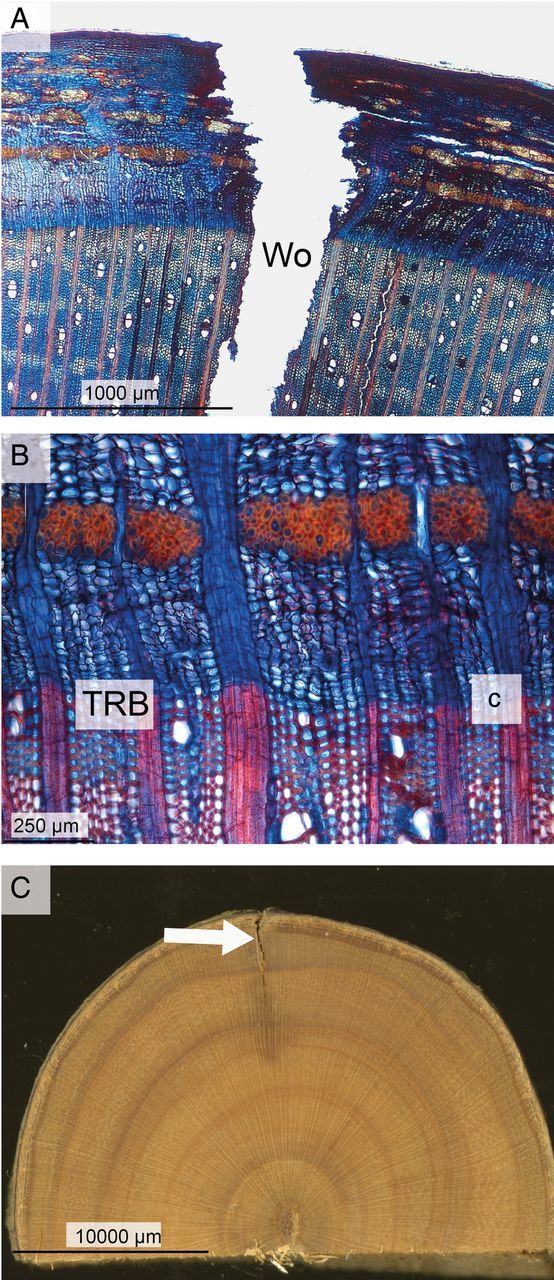
Wound reactions of dormant *A. palmatum* trees after being stored for 3 weeks in a climate chamber at a temperature of 4 °C. (A) Xylem and bark surrounding the wound (Wo). The virtual absence of Cd is characteristic for this cold treatment. (B) Details of the cambial zone near the wound margin, showing the TRB and dormant cambium (c). (C) Transverse section through the wood and bark surrounding the wound (arrowed). Compartmentalization of the wound by inhibitory compounds is virtually absent.

In the living bark, i.e. phloem and cortex, no wound periderms were observed. Nevertheless, during both the winters (*P* < 0.001), we detected the formation of a ligno-suberized layer between the wounded and intact living bark in all the trees subjected to the warm treatment (Fig. 2A). This layer was absent in the trees subjected to the cold treatment. Furthermore, we observed that discolouration in the phloem and cortex was minor in trees subjected to the cold treatment (Fig. 3A), but appreciably greater in trees from the warm treatment (Fig. 2A).

**Figure 4.**
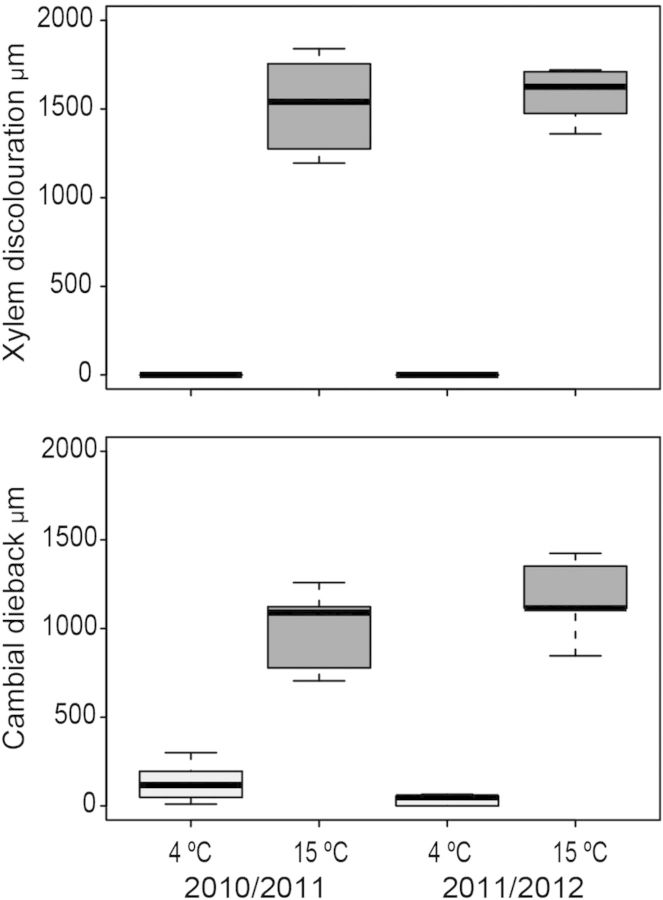
Boxplot showing the extent of tangential xylem discolouration (top) and cambial dieback (bottom) in trees (*n* = 5 per treatment) that were wounded and stored for 3 weeks under either a cold or a warm temperature regime in an experiment conducted during the winters of 2010/2011 and 2011/2012. During both the winters significant (Mann–Whitney *U*, *P* < 0.01) and consistent differences occurred in the extent of xylem discolouration and cambial dieback: at 4 °C hardly any cambial dieback or discolouration was present but at 15 °C cambial dieback and discolouration averaged 1050 and 1550 µm, respectively.

In both winters, the tangential extent of cambial dieback after the 3-week experiments was significantly less (*P* = 0.009) in the cold treatment than in the warm treatment (Fig. 4). In the warm treatment, cambial dieback extended on average 1050 ± 257 µm (mean ± SD, *n* = 10) while in the cold treatment it was virtually absent, with an average value of 84 ± 96 µm (*n* = 10); in two trees from the cold treatment there was no cambial dieback (Fig. 3A). Only in trees stored at 15 °C, few irregularly shaped callus cells had formed near all the wound margins (*P* = 0.001) (Fig. 2B). In that treatment, the cambium of all trees was locally reactivated and formed wound xylem (*P* = 0.001) over a radial distance of ca. 0.7–1.5 mm near the wound margin within 3 weeks of wounding. Within that period, some vessels and fibre cells lignified (Fig. 2C) but others did not (Fig. 2D). Vessels were mostly clustered and were smaller in area than the vessels that had formed during the previous growing season (Fig. 2D). In the 4 °C treatment no wound xylem was formed (Fig. 3A and B).

In the injured xylem, there was a large difference (*P* < 0.001) between the warm and cold treatments in the amount of tangential discolouration on either side of the wound that resulted from the deposition of inhibitory compounds (Figs 2E, 3C and 4). In trees stored under warm conditions, xylem discolouration extended laterally from the wound for an average of 1550 ± 219 µm (mean ± SD, *n* = 10), whereas in trees kept under cold conditions no discolouration occurred (Figs 2E, 3C and 4). Generally, discolouration of cell walls occurred close to the wound, while with increasing distance from the wound and closer to the unwounded xylem, many vessel and fibre lumens were blocked by inhibitory compounds (Fig. 2E and F).

## Discussion

### Callus and local wood formation at 15 °C

When we examined the samples taken 3 weeks after the trees had been wounded during winter bud dormancy, we found, in contrast to our hypothesis, that the formation of callus cells and local formation of wound xylem had already occurred in trees kept at an ambient temperature of 15 °C. To our knowledge, xylem differentiation has never previously been reported in temperate deciduous trees during winter bud dormancy (Begum *et al.* 2013; Copini *et al.* 2014). Begum *et al.* (2007) reported that in unwounded trees kept under warm conditions (locally heated), xylem differentiation occurred only after bud break. This suggests that in our experiment the local formation of wound xylem in *A. palmatum* was triggered by a wound signal that is active only at a higher temperature. The wound signal might comprise hormones such as jasmonates and ethylene, which are important in wound closure (e.g. Seo *et al.* 1997; Bari and Jones 2009; Ursache *et al.* 2013). In horticulture, cell proliferation following grafting has been reported in deciduous trees during dormancy, when grafted stem sections were exposed to temperatures between 24 and 27 °C while the roots and crown were kept at low temperatures (Lagerstedt 1981; Hartmann 2002). This indicates that our findings might be applicable to other deciduous species. In a garden experiment, Copini *et al.* (2014) found that *A. palmatum* trees that had been wounded in October when most leaves had abscised had formed no local wound xylem or callus 14 or 28 days later: in the 14-day period the average temperature was 7.3 °C (±2.7) and in the 28-day period it was 8.2 °C (±3.3). In the same study, *A. palmatum* trees that had been wounded in March, just before the onset of bud burst and tree-ring formation, showed local wood and callus formation around wounds within 4 weeks during which the average temperature was 9.2 °C (±4.1). In addition, local formation of wound xylem was found in trees wounded at the end of August or September when the TRB had formed and the leaves were still fully developed or had begun to acquire their autumn colour (Copini *et al.* 2014). Why were local wound xylem and callus formation for wound closure both absent in October (Copini *et al.* 2014) but present in December under favourable temperature conditions (this study)? A possible explanation is that the trees are in transition from a resting stage (endodormancy) during October to a quiescent stage of dormancy (ecodormancy) in December (Perry 1971; Lang *et al.* 1987; Begum *et al.* 2013).

### Wound periderm formation

In samples from the warm treatment we detected the formation of ligno-suberized layers between the living and wounded bark, which form before the wound periderm develops (Biggs 1986; Trockenbrodt and Liese 1991; Trockenbrodt 1994; Woodward and Pocock 1996). Other studies have shown that wound periderm formation stops in October and resumes during March of the following year (Fink 1999; Copini *et al.* 2014). During the growing season, a ligno-suberized zone can form quickly—within 1–3 weeks—in *Populus tremula*, *Platanus × acerifolia*, *Salix caprea*, *Tilia tomentosa*, *Sorbus aucuparia*, *Acer pseudoplatanus* and *Betula pendula* (Trockenbrodt and Liese 1991; Trockenbrodt 1994; Woodward and Pocock 1996), as happened in our study. To our knowledge, however, there have been no previous reports of ligno-suberized layers being formed during winter dormancy.

### Cambial dieback is temperature dependent

We found that the extent of cambial dieback in response to wounding during dormancy depends on temperature. The virtual absence of cambial dieback in trees stored under cold conditions during dormancy is unexpected, as in an earlier study (Copini *et al.* 2014), cambial dieback in *A. palmatum* trees wounded between March and October, i.e. shortly before, during and after the growing season, always occurred within 2 weeks of wounding. To the best of our knowledge, the absence of cambial dieback after wounding during winter dormancy has never previously been reported. In contrast, studies on wound reactions in *A. pseudoplatanus*, *B. pendula*, *Fagus sylvatica* and *Fraxinus excelsior* have found that more cambial dieback is measured at the end of the growing season after wounding during winter dormancy (December) than after wounding in autumn and spring (Dujesiefken and Liese 1990; Dujesiefken *et al.* 1991). This implies that cambial dieback in *A. palmatum* wounded during winter dormancy is delayed until the temperature rises again. As the extent of cambial dieback is highly correlated with xylem discolouration (Fig. 4) and with discolouration observed in the phloem and cortex, we assume that cambial dieback is a temperature-dependent physiological process associated with xylem and bark discolouration.

### Compartmentalization caused by inhibitory compounds in the xylem

We observed compartmentalization of the wound by deposition of inhibitory compounds in fibre cells and vessel elements only in the trees stored at 15 °C. This is consistent with a finding reported by Schmitt and Liese (1992) for a field experiment: that during winter dormancy fibrillar inhibitory material may be secreted into fibres and vessel elements within 4 weeks of wounding. They found that whereas *B. pendula* was able to continue secretion throughout the winter, in *Tilia americana* there was no further secretion after February or March wounding. When they performed a laboratory experiment in which they stored wood samples at 4 °C in June for 3 weeks, they found that *T. americana* was unable to secrete fibrillar material—which is in line with our results—whereas *B. pendula* was able to secrete inhibitory compounds (Schmitt and Liese 1992). This confirms that compartmentalization by inhibitory compounds is a temperature-dependent physiological process similar to the formation of ligno-suberized layers, callus and wound xylem, which are all delayed at low temperatures during winter dormancy. Given that compartmentalization of tree wounds restricts moisture loss and damage from pathogens (Shigo 1984; Mireku and Wilkes 1989; Pearce 1996; Fink 1999), it thus seems likely that trees in areas with mild winters might cope better with the effects of wounding than trees in areas with cold winters.

## Conclusions

We conclude that *A. palmatum* trees are able to start wound compartmentalization in the living bark, cambial zone and xylem within 3 weeks under mild (15 °C) ambient temperature during winter bud dormancy. Wound reactions entail phloem, cortex and xylem discolouration by the secretion of inhibitory compounds, and the formation of ligno-suberized layers, callus and local wound xylem. At low temperatures, wound reactions, including necrosis of cambial cells, are virtually absent and are likely delayed until the temperature rises again. It therefore seems likely that trees that are wounded during winter dormancy in areas with mild or warm winters will be better able to cope with wounding because, unlike trees in cold environments, they can compartmentalize wounds even during winter dormancy.

## Sources of Funding

This work was supported by the C.T. de Wit Graduate School for Production Ecology and Resource Conservation and the Dutch National Plant Protection Organization of the Ministry of Economic Affairs, Agriculture and Innovation.

## Contributions by the Authors

P.C., J.d.O. and U.S.-K. designed the research; M.D. and P.C. conducted the experiments and analysed the data. All authors interpreted the results and were involved in writing the manuscript.

## Conflicts of Interest Statement

None declared.
